# Phenotypes of Myopathy-related Actin Mutants in differentiated C2C12 Myotubes

**DOI:** 10.1186/1471-2121-8-2

**Published:** 2007-01-16

**Authors:** Friederike S Bathe, Heidi Rommelaere, Laura M Machesky

**Affiliations:** 1 School of Biosciences, Division of Molecular Cell Biology, University of Birmingham, Birmingham B15 2TT, UK; 2 Flanders Interuniversity Institute for Biotechnology (VIB 09) and Department of Biochemistry, Faculty of Medicine and Health Sciences, Ghent University, 9000, Belgium

## Abstract

**Background:**

About 20 % of nemaline myopathies are thus far related to skeletal muscle alpha-actin. Seven actin mutants located in different parts of the actin molecule and linked to different forms of the disease were selected and expressed as EGFP-tagged constructs in differentiated C2C12 mytoubes. Results were compared with phenotypes in patient skeletal muscle fibres and with previous expression studies in fibroblasts and C2C12 myoblasts/myotubes.

**Results:**

Whereas EGFP wt-actin nicely incorporated into endogenous stress fibres and sarcomeric structures, the mutants showed a range of phenotypes, which generally changed upon differentiation. Many mutants appeared delocalized in myoblasts but integrated into endogenous actin structures after 4–6 days of differentiation, demonstrating a poor correlation between the appearance in myotubes and the severity of the disease. However, for some mutants, integration into stress fibres induced aberrant structures in differentiated cells, like thickening or fragmentation of stress fibres. Other mutants almost failed to integrate but formed huge aggregates in the cytoplasm of myotubes. Those did not co-stain with alpha-actinin, a main component of nemaline bodies found in patient muscle. Interestingly, nuclear aggregates as formed by two of the mutants in myoblasts were found less frequently or not at all in differentiated cells.

**Conclusion:**

Myotubes are a suitable system to study the capacity of a mutant to incorporate into actin structures or to form or induce pathological changes. Some of the phenotypes observed in undifferentiated myoblasts may only be *in vitro *effects. Other phenotypes, like aberrant stress fibres or rod formation may be more directly correlated with disease phenotypes. Some mutants did not induce any changes in the cellular actin system, indicating the importance of additional studies like functional assays to fully characterize the pathological impact of a mutant.

## Background

Congenital myopathies include clinical and heterogeneous disorders characterized by skeletal muscle weakness with a range of severity from mild (minor muscle weakness, long-term survival) to severe (neonatal onset, life-threatening) [1]. The presence of protein rods and aggregates in affected skeletal muscle fibres is used to distinguish nemaline and related myopathies from other neuromuscular disorders and to subdivide this pathology into three classes: In actin myopathy (AM) inclusions of excess thin filaments occupy certain areas of the sarcomere. Intranuclear rod myopathy (IRM) is characterized by the presence of rod bodies in the muscle cell nuclei and in nemaline myopathy (NM), protein rods and aggregates are found within the sarcoplasma of skeletal muscle fibres [2]. Mutations in a wide spectrum of genes have been identified to cause NM, AM and IRM, each of them encoding a known component of the sarcomeric thin filament, including nebulin (NEB), troponin (TN), tropomyosin (TM) and actin (ACTA1). About 20 % of NM, AM and IRM are related to skeletal muscle alpha-actin, being the second common cause after mutations in nebulin, and almost 100 mutations in the ACTA1 gene have been identified so far [3,4].

**Figure 1:**
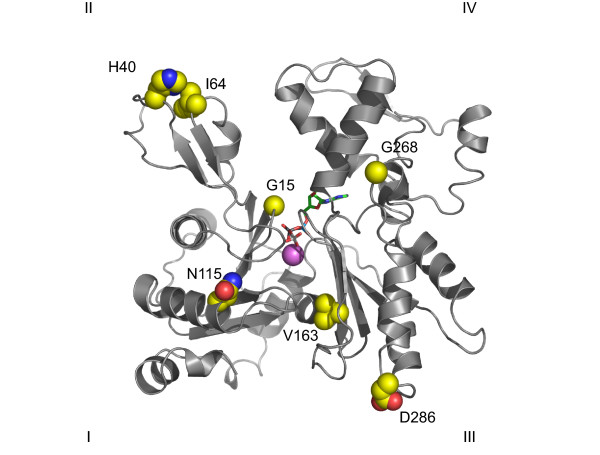
**Position of the seven mutations used in this study on the three dimensional representation of the actin monomer**. The seven mutations selected for this study are represented on the ribbon diagram of the actin molecule from PDB code 1ATN. Calcium is shown in magenta and the adenine of ATP is green with the phosphates in orange. For the side chain atoms, carbon is yellow, nitrogen is blue and oxygen is red.

The seven actin mutants we selected for this study are located in different regions of the actin molecule and represent all three known actin-linked myopathies with varying severity (Figure 1). The G15R-mutation is located in the nucleotide binding pocket and is likely to affect ATP-binding [5]. Like other mutants that affect residues involved in nucleotide binding and turnover, it is linked to actin myopathy (AM) and causes a severe, neonatal life-threatening disorder. H40Y and V163L are located far away from each other within the actin molecule and probably comprise very different functions. However, both of them represent IRM-causing mutants, combined with severe NEM for H40Y and AM for V163L. D286G and G268R are both predicted to affect actin polymerization, since they are situated near the hydrophobic pocket or the hydrophobic plug, respectively, proposed to play an important role in F-actin contacts [6]. The terms hydrophobic pocket and plug refer to the atomic model of the actin filament [6]. Contacts across the two strands in the filament are shown to be mediated in part by a hydrophobic plug consisting of residues 266–269, of one monomer, which inserts across into a hydrophobic pocket formed by residues 166, 169, 171, 173, 285, 289 and 40–45, 63 and 64 of the two adjacent protomers in the opposite strand (see [6]). They are causing severe NEM in patients with profound congenital weakness and high mortality. I64N and N115S are both associated with the milder, typical subtype of NEM. They are located in very different regions, with I64N being at the surface of subdomain II, probably affecting actin polymerization, and N115S in a buried position, likely to affect closure of the nucleotide binding cleft [5].

Some of the selected mutants have been expressed before, either in NIH3T3 fibroblasts [7] or in undifferentiated C2C12 myoblasts [8], using myc-tagged in the former or EGFP-tagged constructs in the latter case. The phenotypes ranged from integration into the endogenous actin network to the appearance of actin accumulations within the cytoplasm or nucleus of transfected cells. For example the IRM-linked actin mutants V163L and H40Y produced nuclear and cytoplasmic aggregates in fibroblasts [7], with a similar result found after expression of EGFP-tagged V163L and V163M mutants in C2C12 myoblasts [8], resembling intranuclear bodies observed in patient muscle samples. Accordingly, two of the NEM-causing actin mutants, D286G and I64N, were found to produce large cytoplasmic rods in fibroblasts, suggested that they may be related to the giant nemaline bodies in patient skeletal muscle fibres [7]. However, other actin isoforms did not show any abnormality in either cell type (G268R, N115 S), raising the question, of whether short-term expression studies in undifferentiated myoblast or fibroblast cell lines are a suitable model to study the effect of myopathy linked actin mutants on the cytoskeletal organization of muscle cells.

The aim of our study was to characterize the behaviour of actin mutants in a differentiated C2C12 myotube system. We expect this to be a better model for myopathy since the contribution of different actin isoforms to the sarcomeric thin filament as the actual functional unit in the muscle can be studied and specific causes of actin dysregulation can be observed. We used C-terminally EGFP-tagged actin constructs, which were transfected into C2C12 cells and myoblasts were then differentiated for 4–6 days. Thus, the mutants reside within the differentiating cells for several days, matching closer the reality in human skeletal muscles where an actin isoform is expressed throughout development.

## Results

### Wild-type EGFP-actin integrated into stress fibres and sarcomeric structures in C2C12 myotubes

We expressed an EGFP-tagged version of wild-type muscle actin (ACTA1) in C2C12 cells to examine its ability to incorporate into endogenous actin structures. In spite of earlier problems regarding expression of actin in differentiated C2C12 cells [7] (C. Costa, doctoral thesis), we were now able to transfect myoblasts and then differentiate them for 4–6 days, using a modified culturing method (see Methods). Myotubes in an advanced developed state were identified by elongated shape, multiple nuclei and a striated staining pattern for alpha-actinin. Alpha-actinin is localized to the Z-discs at an early stage of myogenesis [9,10] and immunostaining for this protein reveals very clearly the sarcomeric structure of striated muscle fibres. Moreover, alpha-actinin has been shown to be component of nemaline bodies [11-13], which are commonly found in muscles of myopathy-patients. In addition, we used fluorescent phalloidin to probe for actin filaments and to test for integration of the transfected actin constructs into endogenous F-actin.

**Figure 2:**
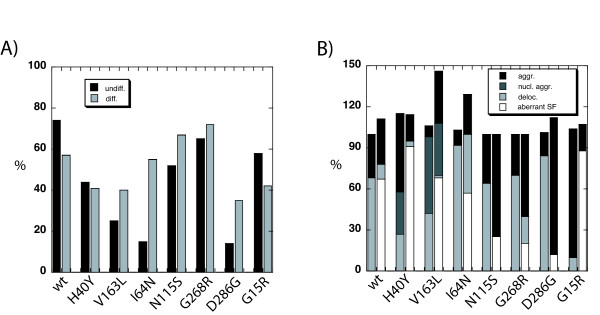
**The phenotypes of actin mutants expressed in C2C12cells differed before and after differentiation**. **A) **Percentages of cells with good integration of the expressed EGFP-tagged actin construct (category 1 cells, see Table 1). Most of the mutant actin isoforms showed better incorporation into the actin cytoskeleton in differentiated myotubes than in undifferentiated cells. **B) **Distribution of different phenotypes of the expressed EGFP-tagged actin constructs in affected C2C12 cells before and after differentiation (category 2 cells, see Table 1). Left bars represent undifferentiated myoblasts, right bars differentiated myotubes. The bars are subdivided, showing the different types of phenotypes that were observed. In some cells, more than 1 phenotype was observed, resulting in total percentages of more than 100%.

**Figure 3:**
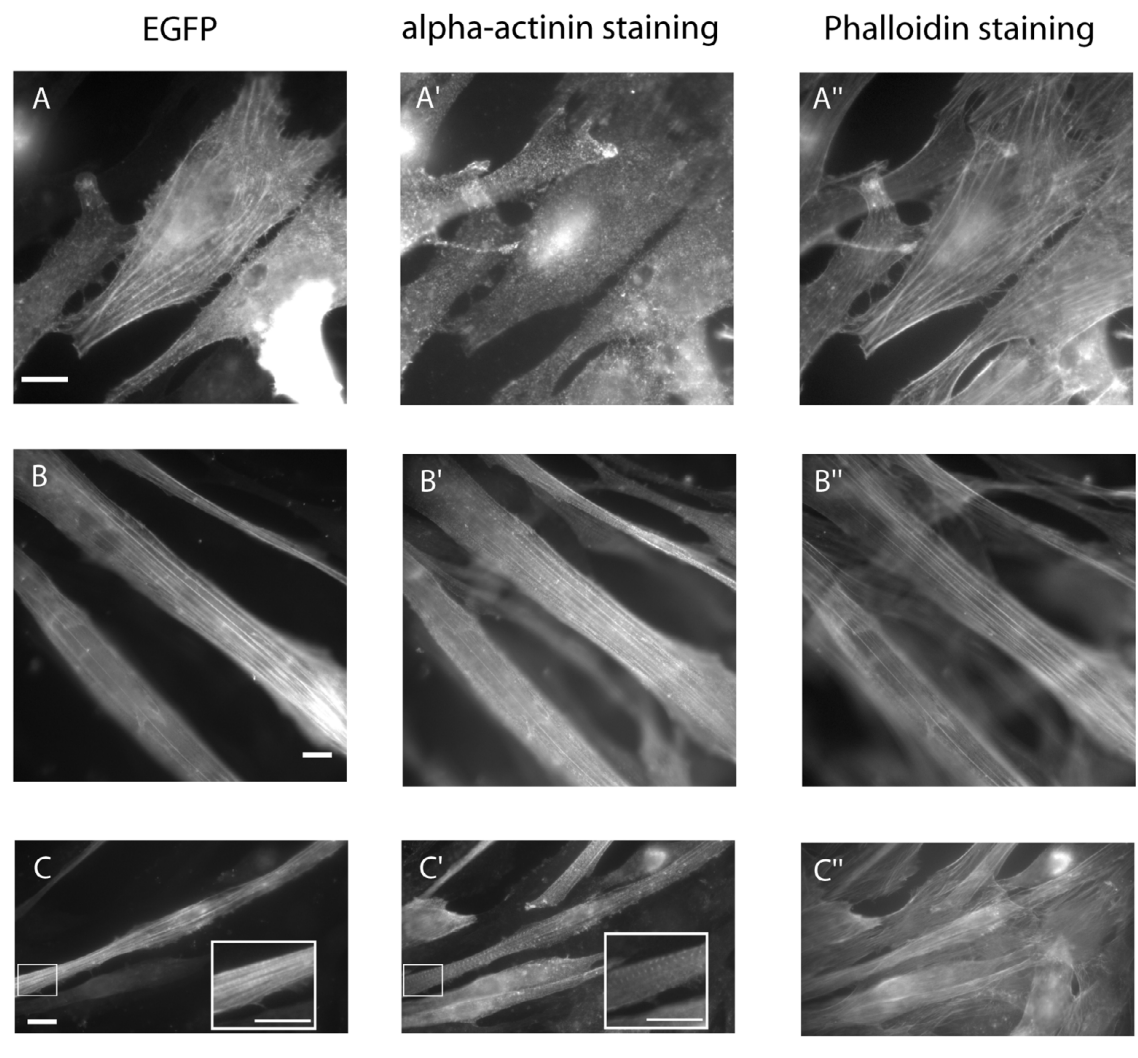
**Expression of wild-type actin-EGFP**. Wild-type muscle actin with a C-terminal EGFP-tag was expressed in undifferentiated C2C12 myoblasts (A) or myotubes that have been differentiated for 4–6 days (B, C). Expressed actin_EGFP co-localized with endogenous filamentous actin, as visualized with phalloidin in myoblasts (A, A") and myotubes (B, B"). In a subset of myotubes, a striated staining-pattern for alpha-actinin revealed sarcomeric structures, with expressed EGFP-actin co-localizing within the sarcomeric thin-filament (C, C"; inset). (Scale bars: 5 μm)

We classified the transfected myoblasts or differentiated myotubes into categories depending on actin incorporation and differentiation state (see Table 1 and legend). In general, the transfection with wild-type or mutant actin did not affect the percentage of cells undergoing differentiation (60–80%, Fig. 2,A). For wt-EGFP-actin the majority of the transfected C2C12 myoblasts showed good integration into stress fibres (75 %, Fig. 3,A); the others containing either small cytosolic aggregates or delocalized EGFP-actin. After 4 to 6 days of differentiation, more than 50 % of the transfected cells were able to differentiate into myotubes, with the EGFP-actin co-localizing with endogenous F-actin (Fig. 3,B). A subset of cells also showed a striated staining pattern, revealing that EGFP-actin localizes within the sarcomeric thin filament (Fig. 3,C; inset). About one third of the transfected cells did not differentiate, the majority of them still showing nice integration of EGFP-actin into stress-fibres. These findings agree with a previous study, where an EGFP-tagged version of muscle actin was found to incorporate into stress fibres and sarcomeric structures in C2C12 cells [8].

**Table 1:** Phenotypes of myopathy-linked actin mutants expressed in C2C12 myoblasts and differentiated myotubes.

	**wt**		**H40Y**		**V163L**		**164N**		**N115S**		**G268R**		**D268R**		**G15R**	
	und.	diff.	und.	diff.	und.	diff.	und.	diff.	und.	diff.	und.	diff.	und.	diff.	und.	diff.
**n**	189	219	174	222	203	189	182	128	52	89	135	127	140	110	114	108
**cat. 1 %**	**74**	**57**	**44**	**41**	**25**	**40**	**15**	**55**	**52**	**67**	**65**	**72**	**14**	**35**	**58**	**42**
**cat. 2 %**	**26**	**3**	**56**	**36**	**75**	**26**	**85**	**5**	**48**	**5**	**35**	**8**	**86**	**44**	**42**	**30**
aggr.	32	33	57	19	8	38	11	29	36	75	30	60	17	100	94	19
nucl.aggr.	0	0	31	0	56	38	0	0	0	0	0	0	0	0	0	0
deloc.	68	11	27	4	42	2	92	43	64	0	70	20	84	0	10	0
aberr. SF	0	67	0	91	0	68	0	57	0	25	0	20	0	12	0	88
**cat. 3 %**		**33**		**5**		**7**		**23**		**22**		**17**		**7**		**11**
**cat. 4 %**		**7**		**18**		**25**		**17**		**2**		**2**		**11**		**18**
aggr.		38		38		35		27		100		100		100		42
nucl.aggr.		0		21		58		0		0		0		0		0
deloc.		56		8		2		83		0		0		0		0
aberr. SF		13		49		40		0		0		0		8		84

Table 1 shows a summary of data from experiments where EGFP-tagged actin constructs were transfected into C2C12 cells and myoblasts were then differentiated for 4–6 days. After immunostaining, transfected myoblasts or differentiated myotubes were classified into categories depending on how well the EGFP-actin was integrated and the differentiation state of the cells:

**myoblasts (und.): category 1**: cells with good integration of EGFP-actin construct

**category 2**: cells with poor integration of EGFP-actin construct, divided into different subgroups: cytoplasmic aggregates (aggr.), nuclear aggregates (nucl. aggr.), delocalization (deloc.), aberrant stress fibres (aberr. SF)

**myotubes (diff.): category 1**: differentiated cells with good integration of EGFP-actin consructs

**category 2**: differentiated cells with poor integration (see subgroups above)

**category 3**: undifferentiated cells with good integration

**category 4**: undifferentiated cells with poor integration (see subgroups above)

For every construct, the total number of transfected cells that were counted is also stated (n).

### IRM-linked actin mutants formed nuclear and cytoplasmic aggregates, but could also integrate into stress fibres, inducing aberrant structures

**Figure 4:**
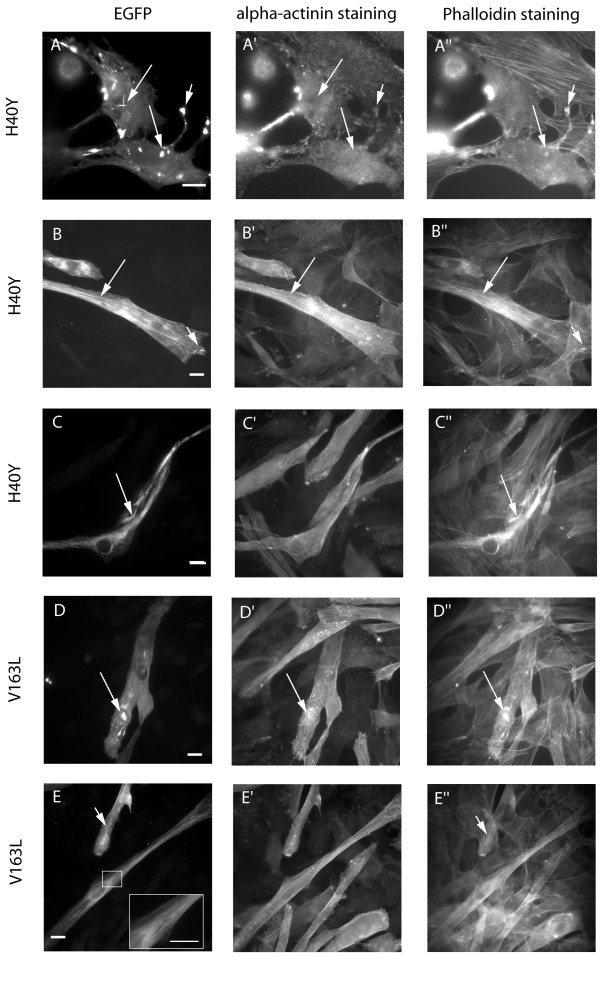
**Expression of the IRM-linked H40Y- and V163L-actin mutants**. EGFP-tagged H40Y- (A-C) and V163L- (D, E) actin mutantswere expressed in C2C12 myoblasts (A) and differentiatedmyotubes.(B-E). EGFP fluorescence showed intranuclear (A; long arrows) and cytoplasmic aggregates (A; short arrow) formed by the H40Y-mutant in undifferentiated cells, with some co-staining with phalloidin (A"; arrows), but not with alpha-actinin (A'; arrows). A similar phenotype was observed for the V163L-isoform (not shown). In myotubes, some integration of EGFP-actin into stress fibres or sarcomeric structures was observed for both, H40Y- actin (B-B"; long arrows) and V163L-actin mutants (E, inset). However, in many differentiated cells, aberrant stress fibres were produced with a thickened and wavy appearance (C, C" long arrows; E, E", short arrows). The H40Y mutant might also induce fragmentation of stress fibres (B, B"; short arrow), but this could also be a short cytoplasmic rod. The V163L-mutant also formed nuclear aggregates in some myotubes (D; long arrow) that co-stained with phalloidin (D"; long arrow) and also very faintly with alpha-actinin (D'; long arrow). Nuclear aggregates were never found with the H40Y-mutant after differentiation. (Scale bars: 5 μm)

In contrast to the wild-type, transfection of the IRM-linked actin mutants H40Y and V163L showed aberrant incorporation in more than 50 % of undifferentiated C2C12 cells (56 % for H40Y, 75 % for V163L, Table 1). Similar to previous studies [7,8], nuclear aggregates have been found in a large subset of those cells, which have been proposed to be related to the intranuclear rods present in skeletal muscles of IRM patients (Fig. 4,A; long arrow). Aggregates showed some co-staining with phalloidin (Fig. 4,A"), indicating the presence of filamentous actin. In almost no case was co-localization of alpha-actinin with aggregates observed, however, in V163L-transfected cells sometimes a very faint co-staining with alpha-actinin could be detected (Fig. 4,D'; long arrow). In contrast to some previous studies [7,8] both mutants were also able to incorporate into stress fibres in a certain portion of C2C12 myoblasts.

After differentiation, 40 % of the transfected myotubes showed good incorporation of mutant actin (Fig. 4,B,E). However, almost half of H40Y-transfected, differentiated cells showed stress fibres with aberrant structures (Table 1). In some cases, stress fibres appeared to be fragmented (Fig. 4,B; short arrow), others were thickened and had a wavy appearance (Fig. 4,C; long arrow). Aberrant stress fibres were also observed in many of the V163L-transfected myotubes (Fig. 4,E; short arrow), but here the phenotypes were more mixed, with some cells showing cytosolic and nuclear aggregates, similar to undifferentiated cells (Fig. 4,D; long arrow). Notably, for V163L, nuclear aggregates were found less frequently in differentiated cells and not at all in H40Y-transfected myotubes, indicating that either cells containing H40Y-actin accumulations in the nucleus stay in an undifferentiated state or nuclear aggregates disappear upon differentiation. Thus, we confirmed the formation of nuclear aggregates being the most striking feature of IRM-linked mutants in myoblasts, however, in differentiated cells aberrant stress fibres became the dominant phenotype.

### Actin mutants linked to typical NEM induced phenotypes in undifferentiated cells, but integrated well into sarcomeric structures in later differentiation states

**Figure 5:**
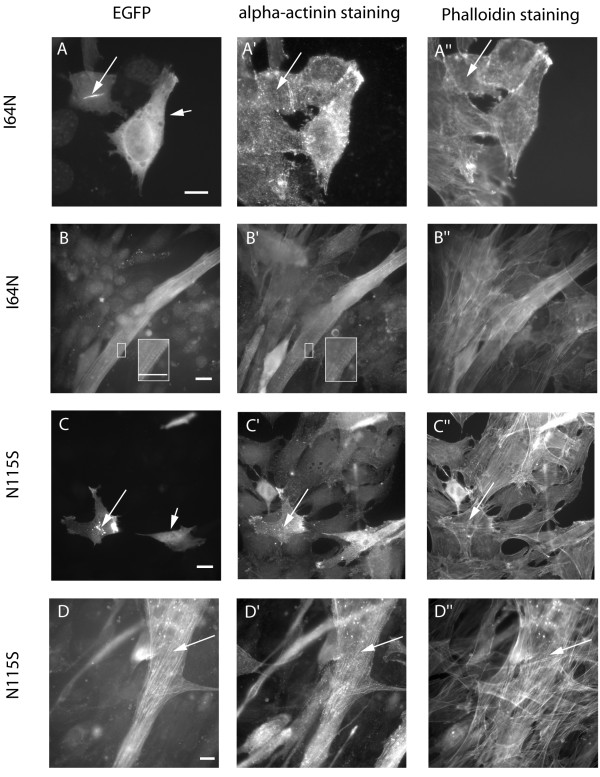
**Expression of the NEM-linked I64N- and N115S-actin mutants**. EGFP-tagged I64N- (A, B) and N115S- (C, D) actin mutantswere expressed in C2C12 myoblasts (A, C) and differentiated myotubes (B, D). Both mutants showed poor integration in undifferentiated cells: EGFP fluorescence revealed delocalization in many myoblasts (A, C; short arrows). I64N-actin also produced large rods (A; long arrow), whereas N115S-actin formed smaller, punctuate aggregates in the cytoplasm of some cells (C; long arrow), in both cases not co-staining with phalloidin or alpha-actinin (A', A"; C', C"; long arrows). After differentiation both mutants incorporated into actin structures such as stress fibres (D-D"; long arrows) and sarcomeric thin filament bundles (B, B', inset). (Scale bars: 5 μm)

Next, we characterized two mutants, N115S- and I64N-actin, that are linked to the generation of NEM in patient muscles. I64N-actin produced diffuse cytoplasmic staining in a large proportion of transfected myoblasts (85 %, Table 1), with some of the cells also showing cytoplasmic rods that did not co-stain with phalloidin or alpha-actinin (Fig. 5,A; long arrow). N115S-actin had a milder effect, but still exhibited poor integration in almost 50 % of the cells, with either delocalization or formation of small cytoplasmic aggregates that did not stain with phalloidin (Fig. 5,C; long arrow). The localization of mutant actins changed considerably upon differentiation. 50 % of the I64N-transfected cells differentiated into myotubes and showed nice integration of the mutant actin into sarcomeric structures, similar to the wild-type (Fig. 5,B; inset). Only a small proportion of differentiated myotubes exhibited phenotypes such as delocalization, aggregates and wavy stress fibres. Most of the cells with such an appearance stayed in an undifferentiated state (Table 1).

Myotubes expressing N115S-actin were even less affected. The majority of them were well differentiated and incorporated the mutant actin nicely into stress fibres and sarcomeric structures (Fig. 5,D). The proportion of cells showing no aberrant structures was even higher than with wt-actin (67 % vs 57 %), indicating no visible defect of this isoform to contribute to endogenous actin structures. Thus, we find that most of the myopathy mutant actins incorporate better in differentiated myotubes than in myoblasts or fibroblasts (Fig. 2,A).

### Two actin mutants that cause a severe form of NEM induced very different phenotypes in differentiated myotubes

**Figure 6:**
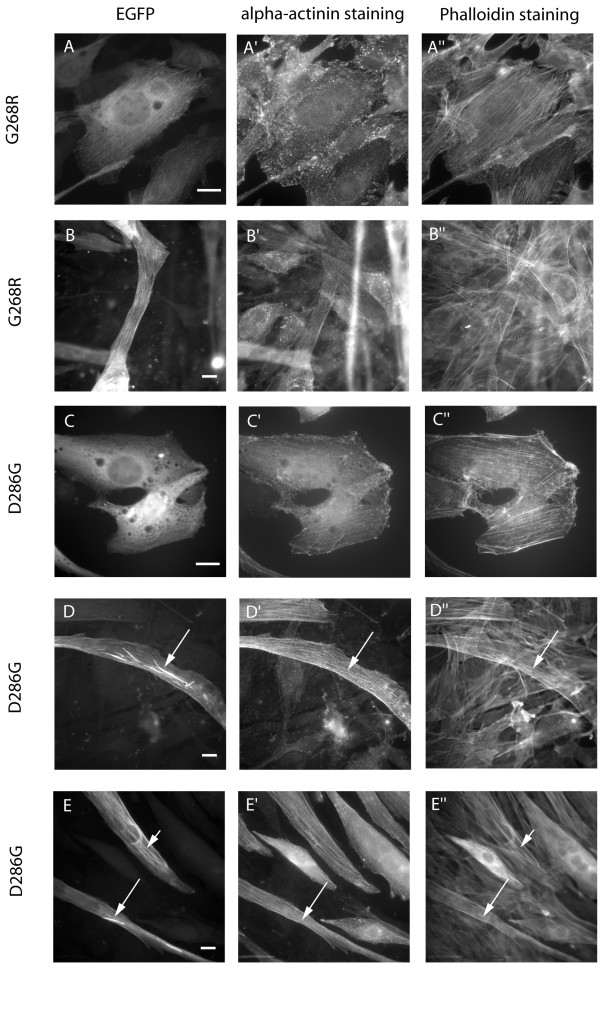
**Expression of the NEM-linked G268R and D286G-actin mutants**. Expression of the EGFP-tagged G268R-actin in either undifferentiated myoblasts (A) or differentiated myotubes (B) lead to good integration of the actin mutant into endogenous actin structures. In contrast, the D286G-isoform stayed delocalized in the majority of transfected myoblasts (C). After differentiation, EGFP fluorescence revealed the formation of giant rods in the cytoplasm of D286G-transfected myotubes (D, E; long arrows) that did not co-stain with phalloidin or alpha-actinin (D', D", E', E"; long arrows). In some myotubes, mutant actin integrated into stress fibres, but their structures were abnormally wavy (E, E"; short arrows).

The mutants D286G-actin and G268R-actin were expected to behave similarly to each other in cultured cells, since they are both predicted to affect actin polymerization and cause severe forms of NEM in patients. However, their phenotypes even in undifferentiated myoblasts were very different. G268R-actin behaved similar to wt-actin with more than 60 % of the cells integrating the mutant into stress fibres (Fig. 6,A). In about one third of transfected cells, small cytosolic aggregates or delocalization of the mutant actin were observed. With D286G-actin, only 14 % of the cells showed normal integration. In almost all of the others, diffuse cytoplasmic staining indicated a delocalized actin mutant (Fig. 6,C), a result very similar to the phenotype of I64N-actin transfected cells (see above).

Upon differentiation, the differences between the two mutants became even more distinct. 44 % of D286G-actin transfected cells differentiated into myotubes that contain huge cytoplasmic rods (Fig. 6,D,E; long arrows) and sometimes additionally showed wavy stress fibres (Fig. 6,E; short arrow). Similar to the I64N-mutant, the rods did not co-stain with phalloidin or alpha-actinin. For G268R-actin a clear majority of myotubes showed incorporation of the mutant into sarcomeric structures (Fig. 6,B), a proportion considerably higher than with wt-actin. In spite of its fatal effects in patients, the G268R-actin isoform thus showed no defects in contributing to sarcomeric actin structures in differentiated muscle cells. Thus, the ability of mutant actin to incorporate into actin structures in differentiated myotubes does not necessarily correlate with the severity of the mutation.

### The AM-linked G15R-actin mutant caused accumulations of polymerized actin in myotubes

**Figure 7:**
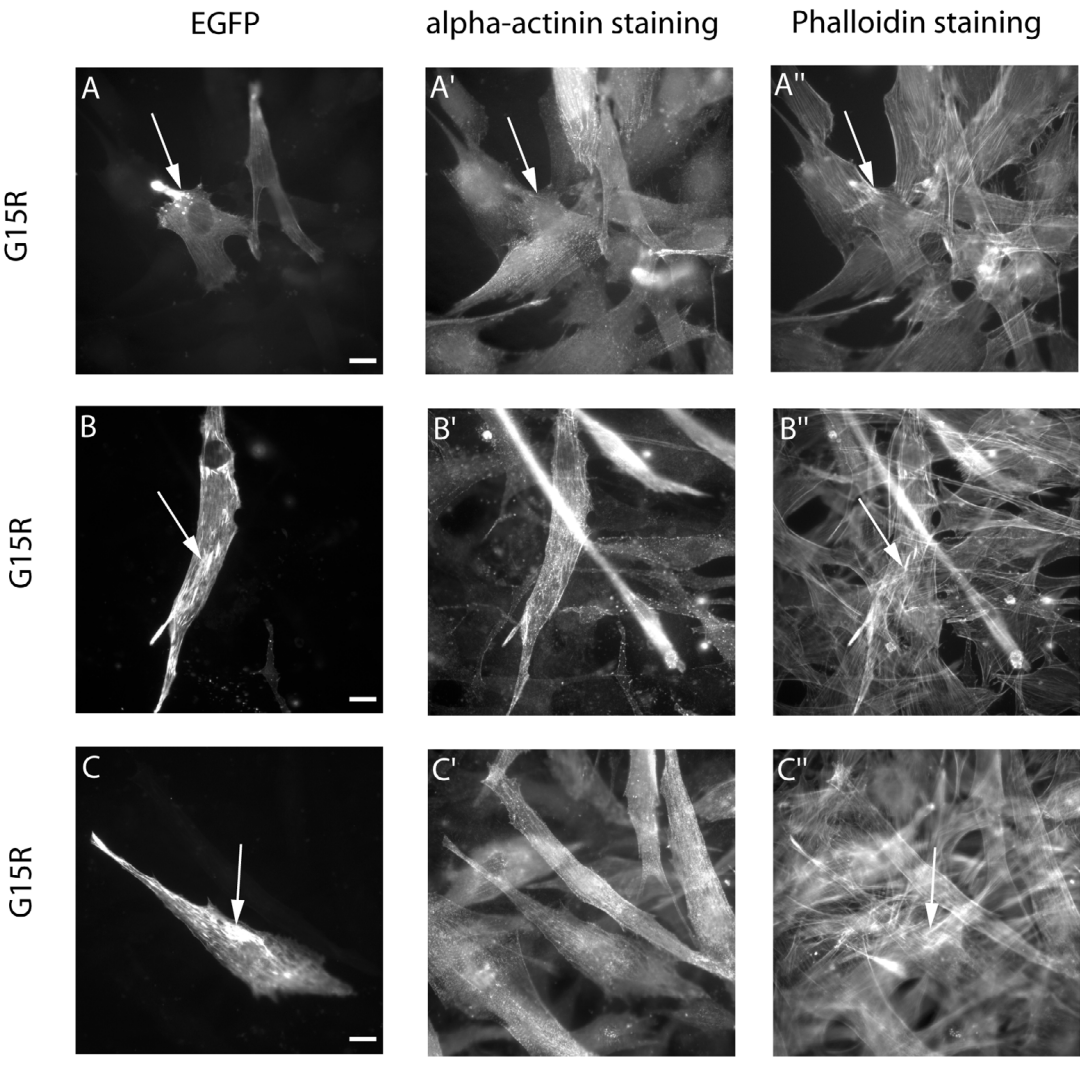
**Expression of the AM-linked G15R-actin mutant**. Expression of the EGFP-tagged G15R-actin isoform in myoblasts produced small cytoplasmic aggregates in many cells (A; long arrow), co-staining with phalloidin but not with alpha-actinin (A', A"; long arrows). In differentiated myotubes, the mutant actin gets integrated, but produced extremely thickened stress fibres (B, B"; long arrows). Sometimes this resulted in big accumulations of polymerized actin that co-stained with phalloidin (C, C"; long arrows).

We also characterized the AM-linked G15R mutant, which is predicted to have defects in ATP-binding. When transfected into undifferentiated myoblasts, many cells had small aggregates in the cytoplasm that mostly stained with phalloidin (Fig. 7,A,A"; long arrows), but more than 50 % also showed good incorporation of the mutant into stress fibres. After differentiation, a more severe phenotype appeared. In one third of the myotubes noticeable changes in actin structures could be observed, especially wavy and extremely thickened stress fibres (Fig. 7,B,C; long arrows). In some cases this resulted in big accumulations of polymerized actin that co-stained with phalloidin and are possibly reminiscent of actin accumulations seen in patient skeletal muscles.

## Discussion

We studied the behaviour of 7 myopathy-related actin mutants in C2C12 myotubes, resulting in a wide range of different phenotypes that in many cases changed during differentiation of the cells. Previous studies have mainly used undifferentiated myoblast or fibroblast cell lines [7,8], although Ilkovski et al. [8] have studied V163L and four other mutants in myotubes. In general, the phenotypes described in these studies matched our findings in the undifferentiated myoblasts, with some differences in how pronounced the effect appeared in the cells (see below).

Notably, none of the mutants affected differentiation, the total proportion of differentiated cells was always at least 60% (the value for wt-actin), for some mutants even as high as 80% (G268R, D286G, Table 1). However, the appearance of most of the mutants investigated differed before and after differentiation (Fig. 2).

Generally, most of the mutant actin isoforms showed better incorporation into the actin cytoskeleton in differentiated myotubes than in undifferentiated myoblasts (Fig. 2,A). This interesting result raises the question, of whether some of the previously described effects are only observed after short-term expression of actin mutants in an *in vitro *cell culture model, but are not relevant for the real situation in skeletal muscles.

The mutants I64N and N115S are not readily integrated into stress fibres, since they produced a diffuse cytoplasmic staining in the majority of undifferentiated myoblasts. For the I64N-mutant this differs slightly from the result by Costa et al., who found some integration into fibroblast stress fibres as well as long cytoplasmic rods [7]. Cytoplasmic rods were also observed in our study in a smaller subset of myoblasts, so we suspect these small differences in appearance may be due to the use of different epitope tags or vectors with somewhat different expression levels (N-terminal myc-tag in Costa et al. and C-terminal EGFP-tag in our study). In case of I64N, poor integraton into stress fibres fits nicely with the predicted polymerization defect [5], also confirmed biochemically in co-polymerization experiments with wt-actin [7]. The N115S mutation is likely to affect the closure of the nucleotide binding cleft, a defect that could also result in a decreased polymerization propensity [5]. However, both isoforms are capable to contribute to sarcomeric structures, since they showed good integration after several days of differentiation (Fig. 5). Only in 5 % of the myotubes an abnormal phenotype could be observed.

Generally, there is poor correlation between the number of myotubes with aberrant actin structures and the severity of the disease caused by a specific mutant. The G268R mutant behaved similarly to the wildtype in undifferentiated myoblasts (Fig. 6,A), confirming the results described in the other expression studies [7,8]. After differentiation, even 72 % of the cells showed good incorporation into actin structures (versus 57% for wt-actin), despite being linked to a severe form of NEM. Because of their pathologically unremarkable phenotype in differentiated myotubes, the NEM-linked mutants I64N, N115S and G268R are likely to be incorporated into sarcomeric structures in patient muscles. Therefore, we suggest that they exert a dominant effect via a functional defect (e.g. contraction defect), rather than mislocalisation. Alternatively, differentiation for 4–6 days might still be not sufficient to show aberrant structures produced by these mutants.

The D286G mutant, which is linked to severe NEM, appeared delocalized in most of the myoblasts (Fig. 6,C), similar to I64N and N115S. However, unlike the other mutants it did not integrate in the majority of myotubes during differentiation, but instead formed big, rod-like aggregates in the cytoplasm (Fig. 6,D,E; long arrows). These rods did not stain with phalloidin, and appeared similar to the aggregates described by Costa et al. in fibroblasts. D286 is located near the hydrophobic pocket, so a profound polymerization defect is likely, which could result in the aggregation of unpolymerized actin. This prediction fits well to the behaviour of the mutant within differentiated cells and to the severity of the disease. It would be interesting to investigate whether patients with this mutation had a higher level of aggregates within muscle biopsies and whether these contained filamentous actin or aggregated actin.

One characterizing feature of NM and also used for diagnostic means is the presence of nemaline rods and bodies in the sarcoplasma of patient skeletal muscles. These giant accumulations are thought to emerge from Z-lines and apart from actin thin filaments are largely composed of alpha-actinin and other Z-line proteins [1,11,13]. Since the transfection of several NEM-linked mutants resulted in the formation of aggregates within the cytoplasm of the cells (Fig. 5, 6), it has been hypothesized that these correspond to the cytoplasmic nemaline bodies found in patient skeletal muscles [7]. However, in our expression studies there are clearly different kinds of accumulations: aggregates that co-stain with phalloidin (V163L, H40Y, G15R), big rods that do not co-stain (I64N, D286G), and small, punctuate aggregates as sometimes found with the wild-type or the phenotypically unremarkable mutants (N115S, G268R), also not co-staining with phalloidin. Aggregates that stain with phalloidin contain actin filaments, as it would be expected in nemaline bodies. The lack of co-staining could either indicate accumulations that are composed of unpolymerized actin or that are not accessible for phalloidin. To further investigate the correlation between the aggregates found in *in vitro *studies and in patient muscles we performed co-staining with alpha-actinin as the main component of nemaline bodies. Generally, there was no co-localization of alpha-actinin with any kind of aggregates formed by the actin mutants, especially the giant rods found with the D286G mutant (Fig. 6,D',E'). Very rarely, some co-staining with aggregates produced by the V163L-isoform was observed (Fig. 4,D'), however, since that occurred only in some cases and was always very faint, the significance of this finding is not clear. The fact that no co-staining was found in the differentiated myotubes is especially interesting, since here early sarcomeric structures including Z-lines have already been constituted, thus providing the original environment for the formation of nemaline bodies. One possible explanation for the lack of co-staining is that the accumulations are not accessible for the antibody. However, we suggest that the propensity of some mutants to form different kinds of rods and aggregates *in vitro *might reflect a specific molecular defect rather than having any connection with the nemaline bodies found in patient muscles. This view is supported by the fact, that the appearance of nemaline rods can vary from 1 % to virtually all fibres and does not correlate with the degree of muscle weakness [14]. Moreover, they are also found in other myopathy diseases as well as in small numbers of normal muscles [1].

A slightly different situation applies for intranuclear rods. In our study, the only mutants that produced intranuclear aggregates are the H40Y and V163L-isoforms, which also cause IRM in patients [15]. This agrees with the study from Ilkovski [8] who found that high levels of insoluble V163L appeared very early after transfection of myoblasts and persisted in myotubes. This indicates that the ability to form accumulations within the nucleus could be an inherent characteristic of some mutants and directly correlated to the appearance of IRM in patients. However, Ilkovski et al. reported nuclear aggregates in myoblasts transfected with the R183G-mutant, which were not found in the muscle biopsy of a patient suffering from severe NEM caused by this isoform [8]. An issue here could be that only limited biopsy material is available from patients, and biopsies tend to be highly variable. Interestingly, nuclear aggregates produced by H40Y-actin in myoblasts and fibroblasts were never observed in differentiated cells, and also did not represent the dominant phenotype in V163L-transfected myotubes (Fig. 2,B), arguing against a direct relation between those aggregates and the rods observed in patient muscles. The molecular reason for the formation of intranuclear rods is still elusive, but a connection has been suggested to the previously reported stress-induced translocation of actin into the nucleus [16,17]. Similar to nemaline bodies, intranuclear rods might be the result of a general response of skeletal muscle fibres to certain pathological situations. Thus, the appearance of intranuclear aggregates *in vitro *might be an indicator for a link to IRM, but cannot be used as an established diagnostic tool.

In addition to rod formation there was another interesting feature predominantly seen in the differentiated myotubes, that is the appearance of aberrant stress fibres. In no case did we observe wavy or otherwise abnormal stress fibres in the myoblasts, but especially for the H40Y-, V163L- and the AM-linked G15R-mutant they were present in a large subset of myotubes (Fig. 2,B). At least the V163L actin also appeared striated, as if incorporated into sarcomere-like structures (Figure 3, inset). The exact phenotype ranges from fragmented and shortened to wavy and thickened stress fibres. The lack of those aberrant structures in undifferentiated cells indicates that they develop after an initial integration of mutant molecules into endogenous actin structures, which eventually induce the formation of abnormal capacities. Fig. 4,B supports this view, since the middle part of the myotube shows normal incorporation of the H40Y-mutant into stress fibres (long arrow), whereas at the far end of the cell, some fragmentation might have occurred (short arrow). Aberrant stress fibres are not seen in undifferentiated cells, but appear for all of the mutants investigated in varying proportions within differentiated myotubes (Fig. 2,B). The percentage of cells containing wavy stress fibres seem to be loosely linked to the severity of the disease, with the highest numbers coming up for the mutants H40Y, G15R and V163L, all causing severe forms of myopathy. A "poisonous" effect of mutant actin isoforms has been previously suggested because of the fact that isoforms were found to be expressed and present within insoluble actin filaments from patient muscles [8] and the recessive nature of "loss-of function" actin mutants with severely defective folding [7].

Unfortunately, we cannot rule out the possibility that differential expression levels among the mutants may contribute to the phenotypes seen. The best system would use the endogenous actin promoter to express mutant actins in cells, but we have not yet succeeded in this aim.

## Conclusion

Differentiated myotubes are a suitable system to characterize myopathy-linked actin mutants. Compared to undifferentiated cells, they show more reliably the capacity of different mutants to incorporate into actin structures or to form or induce pathological changes. The observation of actin isoforms in an environment closer to the reality in skeletal muscles can give hints about which properties of the mutants are likely to cause disease in patients (aberrant stress fibres) and which might be *in vitro *effects nuclear or cytoplasmic aggregates. However, early myotubes are not a model of the situation in skeletal muscles, where additional pathological features may develop as a result of muscular responses (e.g. nemaline bodies). The study of the behaviour of a mutant in myotubes is only one part to fully characterize and predict the impact of an actin mutation, further *in vitro *studies like functional assays or the use of mouse models are also needed.

## Methods

### Construction of the EGFP-tagged alpha-actin myopathy mutants

ACTA1 mutations were made with the Quick Change site directed mutagenesis kit (Stratagene, UK) as described [7]. C-terminal EGFP-tagged wild-type (kind gift from Dr. Nigel Laing) and actin mutants were cloned by PCR using the actin mutants in the pcDNA3.1 vector as template, a 5' primer containing a XhoI site and a 3' primer containing the EcoRI site. These fragments were then ligated into the XhoI/EcoRI-digested pEGFP-N1 vector (Clontech, USA). Constructs were sequenced to verify the complete ACTA1 coding sequence and correct introduction of the desired mutation.

### Cell culture and transfection

C2C12 myoblasts were cultured at 37°C in humidified 5% CO_2 _atmosphere in a proliferation medium composed of Dulbecco's modified Eagle's medium (DMEM 41966, Gibco BRL, UK), supplemented with 10% fetal bovine serum (Sigma, UK).

For expression studies, trypsinized suspensions of C2C12 cells at 0.5 × 10^5 ^cells per ml were seeded on 13-mm glass coverslips coated with gelatine. These were prepared by incubating the coverslips for 5 min in PBS containing 1% gelatine (from bovine skin, Sigma, UK); the gelatine-buffer was then taken off and the coverslips dried for 1 hour under sterile conditions. Alternatively, 13-mm thermanox coverslips (Nalgene Nunc, Naperville, IL) were used.

After 24 h of incubation, the vectors encoding the C-terminally EGFP-tagged actin constructs were transfected using Lipofectamine 2000 transfection reagent (Invitrogen, UK), according to the manufacturer's protocol. Duplicate coverslips were used for every transfected actin construct. After further 24 h incubation, one of the coverslips was taken out and cells were fixed and stained for immunofluorescence (D0, undifferentiated myoblasts). Cells on remaining coverslips were washed twice in differentiation medium, composed of DMEM supplemented with 2% horse serum (Sigma, UK) and then further incubated in this medium for 4–6 days (D4-6, differentiated myotubes).

### Immunofluorescence and microscopy

At given time points, cells plated on coverslips were washed three times in PBS and fixed with 4% formaldehyde for 10 min at room temperature. Free aldehyde groups were blocked with 50 mM NH_4_Cl for 10 min and cells were permeabilized in PBS containing 0.1% Triton X-100 for 4 min. Cells were incubated with anti sarcomeric alpha-actinin (A7811, Sigma, UK) for 20 min at room temperature, followed by a Texas-red anti-mouse secondary antibody (Jackson ImmunoResearch) and Alexa 350-phalloidin (Invitrogen, UK) for 20 min. In some cases, Hoechst staining was applied (0.4 μg/ml) to visualize nuclei and to confirm the presence of intranuclear aggregates. Finally, coverslips were rinsed three times in water and mounted onto a slide with 5 μl Mowiol (Calbiochem) plus Antifade.

Stained cells were examined with a Zeiss microscope (Zeiss, Jena, Germany) using either a 63× or a 100× objective. Images were recorded with a Hamamatsu C4880 camera (Bridgewater, NJ) and processed using Photoshop software (Adobe, USA).

## Authors' contributions

FB carried out all the experiments and drafted the manuscript. HR participated in cloning of the constructs and editing of the manuscript. LMM conceived of the study, participated in its design and edited the manuscript.

## References

[B1] Sanoudou D, Beggs AH (2001). Clinical and genetic heterogeneity in nemaline myopathy--a disease of skeletal muscle thin filaments. Trends Mol Med.

[B2] Clarkson E, Costa CF, Machesky LM (2004). Congenital myopathies: diseases of the actin cytoskeleton. J Pathol.

[B3] Agrawal PB, Strickland CD, Midgett C, Morales A, Newburger DE, Poulos MA, Tomczak KK, Ryan MM, Iannaccone ST, Crawford TO, Laing NG, Beggs AH (2004). Heterogeneity of nemaline myopathy cases with skeletal muscle alpha-actin gene mutations. Ann Neurol.

[B4] Laing NG, Nowak KJ (2005). When contractile proteins go bad: the sarcomere and skeletal muscle disease. Bioessays.

[B5] Sparrow JC, Nowak KJ, Hayley JD, Beggs AH, Walgren-Pettersson C, Romero N, Nonaka I, Laing NG (2003). Muscle disease caused by mutations in the skeletal muscle alpha-actin gene (ACTA1). Neuromuscular Disorders.

[B6] Holmes KC, Popp D, Gebhard W, Kabsch W (1990). Atomic model of the actin filament. Nature.

[B7] Costa CF, Rommelaere H, Waterschoot D, Sethi KK, Nowak KJ, Laing NG, Ampe C, Machesky LM (2004). Myopathy mutations in alpha-skeletal-muscle actin cause a range of molecular defects. J Cell Sci.

[B8] Ilkovski B, Nowak KJ, Domazetovska A, Maxwell AL, Clement S, Davies KE, Laing NG, North KN, Cooper ST (2004). Evidence for a dominant-negative effect in ACTA1 nemaline myopathy caused by abnormal folding, aggregation and altered polymerization of mutant actin isoforms. Hum Mol Genet.

[B9] Dabiri GA, Turnacioglu KK, Sanger JM, Sanger JW (1997). Myofibrillogenesis visualized in living embryonic cardiomyocytes. Proc Natl Acad Sci U S A.

[B10] Sanger JW, Chowrashi P, Shaner NC, Spalthoff S, Wang J, Freeman NL, Sanger JM (2002). Myofibrillogenesis in skeletal muscle cells. Clin Orthop.

[B11] Yamaguchi M, Robson RM, Stromer MH, Dahl DS, Oda T (1982). Nemaline myopathy rod bodies. Structure and composition. J Neurol Sci.

[B12] Morris EP, Nneji G, Squire JM (1990). The three-dimensional structure of the nemaline rod Z-band. J Cell Biol.

[B13] Wallgren-Pettersson C, Jasani B, Newman GR, Morris GE, Jones S, Singhrao S, Clarke A, Virtanen I, Holmberg C, Rapola J (1995). Alpha-actinin in nemaline bodies in congenital nemaline myopathy: immunological confirmation by light and electron microscopy. Neuromuscul Disord.

[B14] Ryan MM, Ilkovski B, Strickland CD, Schnell C, Sanoudou D, Midgett C, Houston R, Muirhead D, Dennett X, Shield LK, De Girolami U, Iannaccone ST, Laing NG, North KN, Beggs AH (2003). Clinical course correlates poorly with muscle pathology in nemaline myopathy. Neurology.

[B15] Kaimaktchiev V, Goebel H, Laing N, Narus M, Weeks D, Nixon R (2006). Intranuclear nemaline rod myopathy. Muscle Nerve.

[B16] Iida K, Matsumoto S, Yahara I (1992). The KKRKK sequence is involved in heat shock-induced nuclear translocation of the 18-kDa actin-binding protein, cofilin. Cell Struct Funct.

[B17] Ono S, Abe H, Nagaoka R, Obinata T (1993). Colocalization of ADF and cofilin in intranuclear actin rods of cultured muscle cells. J Muscle Res Cell Motil.

